# Effectiveness of non-pharmacological interventions on sleep characteristics among adults with musculoskeletal pain and a comorbid sleep problem: a systematic review

**DOI:** 10.1186/s12998-021-00381-6

**Published:** 2021-07-08

**Authors:** Efrosini Papaconstantinou, Carol Cancelliere, Leslie Verville, Jessica J. Wong, Gaelan Connell, Hainan Yu, Heather Shearer, Charlotte Timperley, Chadwick Chung, Bryan J. Porter, Danny Myrtos, Matthew Barrigar, Anne Taylor-Vaisey

**Affiliations:** 1 Faculty of Health Sciences, Ontario Tech University, 2000 Simcoe Street N, Oshawa, ON L1H 7K4 Canada; 2 Centre for Disability Prevention and Rehabilitation, Toronto, Canada; 3 Canadian Memorial Chiropractic College, Toronto, Canada; 4 Institute of Health Policy, Management and Evaluation, University of Toronto, Toronto, Canada; 5 Department of Critical Care, Lakeridge Health, Oshawa, Canada; 6 College of Chiropractic Orthopaedic Specialists (Canada), Toronto, Canada

**Keywords:** Sleep, Musculoskeletal pain, Systematic review

## Abstract

Sleep problems are common and may be associated with persistent pain. It is unclear whether non-pharmacological interventions improve sleep and pain in adults with comorbid sleep problems and musculoskeletal (MSK) pain. We conducted a systematic review on the effectiveness of non-pharmacological interventions on sleep characteristics among adults with MSK pain and comorbid sleep problems. We searched MEDLINE, EMBASE, CINAHL, Cochrane Central and PsycINFO from inception to April 2, 2021 for randomized controlled trials (RCTs), cohort, and case-control studies. Pairs of independent reviewers critically appraised and extracted data from eligible studies. We synthesized the findings qualitatively. We screened 8459 records and identified two RCTs (six articles, 467 participants). At 9 months, in adults with insomnia and osteoarthritis pain, cognitive behavioral therapy for pain and insomnia (CBT-PI) was effective at improving sleep (Insomnia Severity Index, ISI) when compared to education (OR 2.20, 95% CI 1.25, 3.90) or CBT for pain (CBT-P) (OR 3.21, 95% CI 1.22, 8.43). CBP-P vs. education was effective at increasing sleep efficiency (wrist actigraphy) in a subgroup of participants with severe pain at baseline (mean difference 5.45, 95% CI 1.56, 9.33). At 18 months, CBT-PI, CBT-P and education had similar effectiveness on sleep and pain or health outcomes. In adults with insomnia and knee osteoarthritis, CBT-I improved some sleep outcomes including sleep efficiency (diary) at 3 months (Cohen’s d 0.39, 95% CI 0.24, 1.18), and self-reported sleep quality (ISI) at 6 months (Cohen’s d − 0.62, 95% CI -1.01, − 0.07). The intervention was no better than placebo (behavioural desensitization) for improving other sleep outcomes related to sleep onset or pain outcomes. Short-term improvement in sleep was associated with pain reduction at 6 months (WOMAC pain subscale) (sensitivity 54.8%, specificity 81.4%). Overall, in two acceptable quality RCTs of adults with OA and comorbid insomnia, CBT-PI/I may improve some sleep outcomes in the short term, but not pain outcomes in the short or long-term. Clinically significant improvements in sleep in the short term may improve longer term pain outcomes. Further high-quality research is needed to evaluate other non-pharmacological interventions for people with comorbid sleep problems and a range of MSK conditions.

**Supplementary Information:**

The online version contains supplementary material available at 10.1186/s12998-021-00381-6.

## Introduction

Musculoskeletal (MSK) conditions are leading causes of disability worldwide [1]. MSK conditions are the most common causes of severe long-term pain and are typically characterized by limitations in mobility, dexterity and functional ability affecting social functioning and mental health, further diminishing overall quality of life [1]. Further to this, sleep disturbances or problems are frequently experienced by individuals suffering from MSK conditions, but are often seen as simply a symptom of pain, and not as an independent problem [2].

Sleep disturbances include poor quality sleep, reduced sleep efficiency and duration, delayed sleep onset, fragmentation of sleep architecture or sleep continuity, increased activity or movement during sleep, nonrestorative sleep and increased sleepiness during daytime [3, 4]. Sleep problems, such as insomnia and poor sleep quality are amongst the most common comorbidities associated with various MSK conditions [5–8]. In adults with chronic low back or neck pain, the estimated prevalence of sleep problems is approximately 58.7% [9–11]. A prevalence of sleep deprivation (42.2%) has been reported in patients with chronic lower back and neck pain with 19.8% of these patients also reporting serious sleep impairments such as fewer than four hours of sleep per night [10]. The prevalence of insomnia is twice larger in patients with osteoarthritis (OA) (25%) than without (11%). More than two-thirds of patients with OA have sleep disturbances; sleep disturbances have also been found to be a contributing factor to limitations in daily functioning [11]. While chronic pain and chronic insomnia exacerbate profound negative consequences individually, when they co-occur, their combined impact in lost productivity and personal suffering is magnified. Compared to those experiencing only chronic pain, those that experience both chronic pain and difficulties with sleep report higher pain intensity, more depressive symptoms, and greater distress [2, 12–15].

Sleep and pain share a complex reciprocal relationship, such that pain disturbs sleep continuity and quality and, conversely, poor sleep can exacerbate pain intensity. Individuals with MSK pain are prone to suffer from poor sleep, for example, the physical discomfort associated with pain may disrupt sleep by increasing a person’s level of internal arousal. Furthermore, poor sleep may disrupt various physiological processes that can then affect pain perception negatively and can worsen pain by influencing pain signal processing, pain threshold, inflammation, and disability. Disrupted sleep may contribute directly to increased central pain processing, exacerbating daily pain, which creates a vicious cycle of perpetuated sleep disturbances and pain. While an association between pain and sleep problems has been established, this does not demonstrate causality; however, longitudinal studies conducted in both adolescent and adult pain populations have found sleep problems to be predictive of pain suggesting a unilateral relationship.

The sleep-pain relationship is multifactorial and therefore a multi-pronged approach should be taken when managing MSK pain. MSK conditions are typically managed by primary care and rehabilitation professionals such as general physicians, physiotherapists and chiropractors. Evidence-based guidelines recommend that clinicians use a biopsychosocial approach to manage patients with MSK conditions, including screening for and addressing comorbidities and suboptimal lifestyle behaviours. Rehabilitation clinicians, however, typically do not comprehensively assess sleep problems or sleep outcomes [16], despite perceiving sleep as important for health and rehabilitative outcomes [17]. Given that sleep problems are strongly related to pain, and many patients have identified improved sleep as an important outcome for pain treatment this is one area deserving more attention in MSK pain populations.

Two systematic reviews and meta-analyses of randomized controlled trials (RCT) examined the effect of sleep interventions on improving sleep and pain [18, 19]. Tang et al. (2015) reported that non-pharmacological sleep treatments (CBT-I, behavioral therapy, energy, and sleep enhancement) in chronic pain patients were associated with a large improvement in sleep quality and a small reduction in pain at post-treatment for both cancer and non-cancer pain patients. Ho et al. (2019) reported that sleep interventions (e.g., CBT and pharmacological interventions) improved sleep and pain for people with low back pain, and only sleep for people with OA. These systematic reviews require updating, as their literature searches ended in 2014 and 2017, respectively. Further, systematic reviews that seek to identify analytic observational studies (i.e., cohort and case-control studies) in addition to RCTs may add additional knowledge.

To our knowledge, no recent systematic reviews have been conducted to assess the effectiveness of non-pharmacological interventions on sleep and health outcomes including pain (defined by the World Health Organization (WHO) International Classification of Functioning, Disability and Health (ICF) framework) in a population with MSK pain and comorbid sleep problems [20]. Therefore, we conducted a systematic review of the literature to synthesize the best evidence on the effectiveness of non-pharmacological interventions on sleep characteristics among adults with MSK pain and a comorbid sleep problem.

## Materials and methods

Our systematic review protocol was registered with the International Prospective Register of Systematic Reviews (PROSPERO) (#CRD42019130698). We used the Preferred Reporting Items for Systematic Reviews and Meta-Analyses (PRISMA) statement to guide the conduct and reporting of this review [21]. Prior to the analyses, we amended our protocol to clarify that secondary or subsequent analyses of randomized trials were eligible to capture all relevant literature.

### Eligibility criteria

We selected studies based on our predefined inclusion and exclusion criteria.

**Table:** 

***Population***	
Adults (aged 18 years and older) with MSK pain and a comorbid sleep problem	
*Musculoskeletal (MSK) Pain*	
MSK pain involving the soft tissues of the muscles and joints including, but not limited to, non-specific neck, mid-back, low back pain with or without symptoms of radiculopathy, MSK chest pain, cervicogenic headache, tension-type headache, temporomandibular joint pain, MSK extremity pain, and osteoarthritis. Excluded: MSK pain associated with major, structural, systemic pathology (e.g., cancer, osteoporosis, inflammatory arthritis (e.g., ankylosing spondylitis), fractures, dislocations, grade III sprains/strains, infections), or fibromyalgia^a^.	
*Sleep Problems*	
1. Self-reported sleep problems. Common terms and descriptions include, but are not limited to: • Insomnia • Difficulty falling asleep (commonly measured by sleep onset latency [SOL]) • Difficulty maintaining sleep (commonly measured by frequent awakenings, and how long it takes to fall back to sleep after being awoken; also referred to as Wake After Sleep Onset [WASO]) • Awakening too early with the inability to return to sleep • Non-restorative sleep (commonly measured with Pittsburgh Sleep Quality Index [PSQI] or with degree of daytime impairments (sleepiness) such as the Epworth Sleepiness Scale [ESS]) 2. Insomnia Disorder: as defined by DSM-IV, DSM-V or other diagnostic classifications Excluded: all other diagnosed sleep disorders, including but not limited to, sleep-related breathing disorders (sleep apnea, obstructive sleep apnea [OSA], obstructive breathing disorders), central disorders of hypersomnolence (e.g., narcolepsy, hypersomnia), circadian rhythm sleep disorders, parasomnias (sleep walking, sleep terrors, sleep-related eating disorder), and sleep-related movement disorders (restless leg syndrome).	
***Interventions***	
Non-pharmacological interventions including but not limited to: 1. Environmental (e.g., light therapy, earplugs, alarm modifications, headphones, white noise, social support) 2. Behavioral (e.g., CBT and single elements of CBT such as sleep restriction), sleep hygiene education, massage, acupressure and relaxation interventions (e.g., music therapy and guided imagery) 3. Physical therapy (e.g., mobility/exercise during the day to improve sleep at night, acupuncture) 4. Multimodal interventions: sleep interventions combined with other interventions (e.g., sleep intervention combined with an intervention explicitly stated to improve pain) Excluded: any prescription and over-the-counter pharmacological therapies, herbal and dietary sleep supplements including, but not limited to, oral capsules/pills, patches, sprays, drops, and other liquids (e.g., benzodiazepines, non-benzodiazepine pills, antidepressants). Examples of over-the-counter aids include diphenhydarmine (i.e., Nytol, Sominex), and doxylamine (i.e., Unisom, Nighttime Sleep Aid). Dietary sleep supplements include, but were not limited to, valerian, melatonin, chamomile, tryptophan, and kava. Pharmacological interventions combined within a multimodal non-pharmacological approach were considered. We excluded any invasive interventions such as injections and surgeries.	
***Comparison***	
Other interventions (including pharmacological interventions), placebo or sham interventions, wait list, or no intervention.	
***Outcomes***	
Studies evaluating at least one sleep outcome and may have also evaluated a health outcome. Sleep outcomes include: 1) sleep disturbances (difficulty initiating or maintaining sleep, reduced sleep efficiency, altered sleep architecture); 2) sleepiness (difficulty remaining awake); 3) sleep patterns; 4) sleep fragmentation (sleep cycle unable to reach stage 4 non-rapid eye movement [NREM] and rapid eye movement [REM] due to waking up throughout sleep); and 5) self-reported sleep quality (tiredness upon waking, daytime tiredness, feelings of being rested and restored). Common self-reported measures of sleep include the Pittsburgh Sleep Quality Index (PSQI) [22], and Insomnia Severity Index (ISI) [23]; objective measures include wrist actigraphy [24]. Health outcomes were classified according to the WHO International Classification of Functioning, Disability and Health (ICF) framework: 1) body function and structure (e.g., pain intensity, depression, anxiety), and 2) activity and participation (e.g., communication, mobility, interpersonal interactions, self-care, learning, applying knowledge, return to work/activities/school). Common measures of pain include the visual analogue scale (VAS), numerical rating scale (NRS), and McGill Pain Questionnaire. We also included health-related quality of life (e.g., SF-12)	
***Studies***	
1. English language 2. Published in a peer-reviewed journal 3. RCT with minimum 30 participants per arm at baseline^b^ 4. Cohort and case-control studies with minimum 100 participants per group at baseline^c^ 5. Secondary analyses of eligible RCTs, cohort and case-control studies Excluded: cross-sectional studies, case reports, case series, pilot studies, study protocols, qualitative studies, non-systematic and systematic reviews, clinical practice guidelines, biomechanical studies, laboratory studies, cadaveric or animal studies, guidelines, letters, editorials, commentaries, unpublished manuscripts, dissertations, government reports, books and book chapters, conference proceedings, meeting abstracts, lectures and addresses, consensus development statements, guideline statements.	

^a^Fibromyalgia was excluded due to its clinical presentation of chronic widespread pain, fatigue and sleep disturbance symptoms (DSM-10). This condition may not be appropriately managed by the sleep interventions identified in this review

^b^A sample size of 30 per arm in RCTs is conventionally considered the minimum needed for non-normal distributions to approximate the normal distribution [25]. The assumption that data is normally distributed is required to ascertain a difference in sample means between treatment arms

^c^A sample of 100 is conventionally considered the minimum needed to obtain well-balanced groups at baseline and control bias [25].

### Information sources

We developed our search strategy in consultation with a health sciences librarian, and a second librarian reviewed the search for completeness and accuracy using the Peer Review of Electronic Search Strategies (PRESS) Checklist [26]. We searched MEDLINE, Embase, CINAHL, Cochrane Central Register of Controlled Trials and PsycINFO from inception to April 2, 2021.

The search strategy was first developed in MEDLINE and subsequently adapted to the other databases (see supplementary material). The search terms included subject headings specific to each database (e.g., MeSH in MEDLINE) and free text words relevant to non-pharmacological interventions, sleep disturbances, and MSK pain. We used EndNote X9 software to create a database containing the search results.

### Study selection

Pairs of trained, independent reviewers screened articles in two phases to determine eligibility. In phase I, paired reviewers screened titles and abstracts to determine possibly relevant and irrelevant citations based on the outlined inclusion and exclusion criteria. In phase II, paired reviewers reviewed possibly relevant citations from the first phase using the full text article to determine eligibility. Any disagreements during screening were resolved by discussion between the paired reviewers to reach consensus. If consensus could not be reached, a third reviewer independently appraised the citation and discussed with the other two reviewers to reach consensus.

### Quality assessment

Pairs of trained, independent reviewers critically appraised all relevant studies using the Scottish Intercollegiate Guidelines Network (SIGN) criteria for controlled trials, cohort studies, and case-control studies [27]. Consensus between reviewers was reached through discussion with the involvement of an independent third reviewer where necessary. We contacted authors when additional information was needed to complete the appraisal. A study was considered to have a high risk of bias if reviewers considered that the study’s internal validity was compromised as a result of biases and methodological flaws.

### Methodological aspects critically appraised by study design

**Table:** 

**RCT** 1) clarity of the research question, 2) randomization method, 3) concealment of treatment allocation, 4) blinding of treatment and outcomes, 5) similarity of baseline characteristics between treatment arms, 6) co-interventions/contamination, 7) validity and reliability of outcome measures, 8) attrition, 9) intention to treat analysis, and 10) comparability of results across study sites (if applicable).	
**Cohort** 1) clarity of the research question, 2) comparability of groups, 3) participation rates, 4) population at risk, 5) attrition rates, 6) analysis of missing follow-up data, 7) clearly defined outcomes, 8) blinding of outcome assessor, 9) assessment of impact on outcome assessment with knowledge of exposure, 10) reliable assessment of exposure, 11) validity and reliability of outcome measures, and 12) repeated measures of exposure level or prognostic factor.	
**Case-control** 1) clarity of the research question, 2) comparability of populations between cases and controls, 3) similarity of exclusion criteria used for cases and controls, 4) participation rates, 5) comparability between participants and non-participants, 6) clarity of differentiation between cases and controls, 7) certainty that controls are non-cases, 8) knowledge of exposure did not influence case ascertainment, 9) validity and reliability of exposure status, and 10) handling of potential confounders.	

### Data extraction

Pairs of independent reviewers extracted the data from each eligible study to create the evidence table (Table 1). Consensus between reviewers was reached through discussion with the involvement of an independent third reviewer where necessary. We extracted: 1) author, year and country; 2) MSK condition; 3) sleep problem criteria; 4) participant characteristics; 5) intervention arms; content; delivery, dosage and duration; 6) outcomes; and 7) key findings. Where multiple outcome measures were used to assess each construct, we extracted data for all measures.

**Table 1:** Included studies: *n* = 6 studies (representing 2 randomized controlled trials)

Author, year, country, study design	Musculoskeletal condition and participants	Sleep problem criteria	Intervention arms	Intervention content	Intervention delivery, dosage, duration	Outcome measures	Key findings
Vitiello et al. 2013 [31] USA Lifestyles RCT (9-mo FU)	Osteoarthritis Grade II to IV pain on Graded Chronic Pain Scale (GCPS) N = 367 78% female Mean age = 73y (SD 8.2)*	DSM-IV-TR for insomnia: self-reported sleep difficulties ≥3 nights/week during past month with ≥1 daytime sleep related problem	CBT pain and insomnia (CBT-PI) (n = 122) CBT pain (CBT-P) (*n* = 122) Education only control (EOC) (*n* = 123)	CBT pain as below and standard CBT for insomnia (sleep hygiene education, stimulus control, sleep restriction, daily sleep monitoring). Pain education, physical education, goal setting, relaxation, activity pacing, guided imagery, cognitive restructuring). Educational content related to pain and sleep management. Classes facilitated in nondirective, self-help format.	All interventions delivered as group interventions by mental health professionals; 90-min group sessions (5–12 individuals) 1x/week, for 6 weeks	**Sleep** ***Insomnia severity:*** *Insomnia Severity Index (ISI):* 0–28 (lower is better) MCID: 3.45 (defined as 30% reduction from baseline) ***Sleep efficiency (SE):*** *wrist actigraphy* (over 1 week): 0–100% (higher is better) MCID: 5%* **Pain** ***Pain severity:*** *Graded Chronic Pain Scale (GCPS):* 0–10 (lower is better) MCID: 1.3 (defined as 30% reduction from baseline) ***Arthritis symptoms:*** *Arthritis Impact Measurement Scale V2 (AIMS):* 1–10 (lower is better) MCID: 1.8* (defined as 30% reduction from baseline)	9-mo FU **Treatment effect estimate [95%CI]:** CBT-P vs. EOC ISI: 0.13 [− 0.89,1.16] (no statistically significant difference) GCPS: 0.08 [− 0.21, 0.38] (no statistically significant difference) SE: 2.91% [0.85, 4.97] (non-clinically important difference favouring CBT-P) AIMS: − 0.06 [− 0.39, 0.28] (no statistically significant difference) CBT-PI vs. EOC ISI: − 1.89 [− 2.83, − 0.96] (non-clinically important difference favouring CBT-PI) GCPS: − 0.09 [− 0.37, 0.18] (no statistically significant difference) SE: 2.64% [0.44, 4.84] (non-clinically important difference favouring CBT-PI) AIMS: 0.20 [− 0.26, 0.66] (no statistically significant difference) CBT-PI vs. CBT-P ISI: − 2.03 [− 3.01, − 1.04] (non-clinically important difference favouring CBT-PI) SE: − 0.26% [− 2.82, 2.29] (no statistically significant difference) **Clinically significant treatment effect (OR [95% CI]):** CBT-P vs. EOC ISI: 0.81 (0.48, 1.36) (no statistically significant difference) GCPS: 0.79 (0.39, 1.60) (no statistically significant difference) CBT-PI vs. EOC ISI: 2.20 (1.25, 3.90) (clinically important difference favouring CBT-PI) GCPS: 0.96 (0.55, 1.68) (no statistically significant difference) CBT-PI vs. CBT-P ISI: 2.72 (1.59, 4.64) (clinically important difference favouring CBT-PI) **Treatment effect estimate [95%CI] for participants with severe pain at baseline:** CBT-P vs. EOC ISI: − 0.29 (− 2.36, 1.77) (no statistically significant difference) GCPS: − 0.16 (− 0.68, 0.37) (no statistically significant difference) SE: 5.45% (1.56, 9.33) (clinically important difference favouring CBT-P) AIMS: 0.05 (− 0.60, 0.69) (no statistically significant difference) CBT-PI vs. EOC ISI: − 2.71 (− 4.91, − 0.51) (non-clinically important difference favouring CBT-PI) GCPS: − 0.44 (− 1.00, 0.11) (no statistically significant difference) SE: 3.69% (0.72, 6.66%) (non-clinically important difference favouring CBT-PI) AIMS: 0.28 (− 0.55, 1.10) (no statistically significant difference) CBT-PI vs. CBT-P ISI: − 2.42 (− 4.15, 0.68) (no statistically significant difference) SE: − 1.76% (− 6.10, 2.58%) (no statistically significant difference) **Clinically significant treatment effect (OR [95% CI]) for participants with severe pain at baseline:** CBT-P vs. EOC ISI: 0.75 (0.27, 2.07) (no statistically significant difference) GCPS: 1.18 (0.49, 2.85) (no statistically significant difference) CBT-PI vs. EOC ISI: 2.41 (0.93, 6.21) (no statistically significant difference) GCPS: 1.36 (0.57, 3.24) (no statistically significant difference) CBT-PI vs. CBT-P ISI: 3.21 (1.22, 8.43) (clinically important difference favouring CBT-PI)
McCurry et al. 2014 [42] USA Lifestyles RCT (18-mo FU)							18-mo FU **Treatment effect estimate [95%CI]:** CBT-P vs. EOC ISI: 0.32 [− 0.97, 1.61] (no statistically significant difference) GCPS: − 0.04 [− 0.53, 0.45] (no statistically significant difference) SE: 0.91% [− 2.10, 3.91%] (no statistically significant difference) AIMS: − 0.09 [− 0.63, 0.45] (no statistically significant difference) CBT-PI vs. EOC ISI: − 0.86 [− 2.13, 0.40] (no statistically significant difference) GCPS: − 0.36 [− 0.82, 0.10] (no statistically significant difference) SE: 2.10% [− 1.39, 5.59%] (no statistically significant difference) AIMS: 0.07 [− 0.55, 0.68] (no statistically significant difference) CBT-P vs. CBT-PI* ISI: − 0.53 [− 3.08, 2.02] (no statistically significant difference) GCPS: − 0.54 [− 1.63, 0.55] (no statistically significant difference) SE: 2.59% [− 4.63, 9.81%] (no statistically significant difference) AIMS: − 0.01 [− 1.16, 1.15] (no statistically significant difference) **Clinically significant treatment effect (OR [95% CI]):** CBT-P vs. EOC ISI: 1.06 (0.59, 1.90) (no statistically significant difference) GCPS: 1.05 (0.52, 2.13) (no statistically significant difference) CBT-PI vs. EOC ISI: 1.51 (0.72, 3.12) (no statistically significant difference) GCPS: 1.21 (0.52, 2.82) (no statistically significant difference) **Treatment effect estimate [95%CI]) for participants with severe pain at baseline:** CBT-P vs. EOC ISI: 0.45 (− 1.84, 2.74) (no statistically significant difference) GCPS: 0.09 (− 0.78, 0.96) (no statistically significant difference) SE: 1.75% (− 2.36, 5.86) (no statistically significant difference) AIMS: 0.14 (− 0.88, 1.16) (no statistically significant difference) CBT-PI vs. EOC ISI: − 1.63 (− 3.77, 0.50) (no statistically significant difference) GCPS: − 0.55 (− 1.48, 0.39) (no statistically significant difference) SE: 2.53% (− 3.29, 8.35) (no statistically significant difference) AIMS: 0.42 (− 0.69, 1.52) (no statistically significant difference) **Clinically significant treatment effect (OR [95% CI]) for participants with severe pain at baseline:** CBT-P vs. EOC ISI: 0.59 (0.17, 2.11) (no statistically significant difference) GCPS: 1.01 (0.35, 2.92) (no statistically significant difference) CBT-PI vs. EOC ISI: 2.06 (0.51, 8.41) (no statistically significant difference) GCPS: 1.64 (0.40, 6.80) (no statistically significant difference)
Vitiello et al. 2014 [41] USA Secondary analysis of Lifestyles RCT						**Sleep** ***Insomnia severity:*** *Insomnia Severity Index (ISI):* 0–28 (lower is better) MCID: 3.47 (defined as 30% reduction from baseline) ***Sleep efficiency (SE):*** *wrist actigraphy* (over 1 week): 0–100% (higher is better) MCID: 5%* ***Sleep quality:*** *Pittsburgh Sleep Quality Index (PSQI):* 0–21 (lower is better) MCID: 3* ***Sleep beliefs and attitudes:*** *Dysfunctional Beliefs and Attitudes About Sleep Scale (DBAS-10):* 0–100 (lower is better) MCID: 14.6* (defined as 30% reduction from baseline) ***Fatigue:*** *Flinders Fatigue Scale (FFS):* 0–31 (lower is better) MCID: 3.4* (defined as 30% reduction from baseline) ***Daytime sleepiness:*** *8-item Epworth Sleepiness Scale (ESS):* 0–24 (lower is better) MCID: 2* ***Daytime function:*** *10-item Functional Outcomes of Sleep Questionnaire (FOSQ-10):* 5–20 (higher is better) MCID: 5.22* (defined as 30% higher from baseline) **Pain** ***Pain severity:*** *Graded Chronic Pain Scale (GCPS):* 0–10 (lower is better) MCID: 1.3 (defined as 30% reduction from baseline) ***Arthritis symptoms:*** *Arthritis Impact Measurement Scale V2 (AIMS):* 1–10 (lower is better) MCID: 1.8* (defined as 30% reduction from baseline) ***Catastrophizing:*** *Pain Catastrophizing Scale (PCS):* 0–52 (lower is better) MCID: 3.36* (defined as 30% reduction from baseline) ***Fear avoidance:*** *Tampa Scale for Kinesiophobia (TSK):* 17–68 (lower is better) MCID: 4* ***Depression:*** *Geriatric Depression Scale (GDS):* 0–30 (lower is better) MCID: 2.1* (defined as 30% reduction from baseline) Improvers: ≥30% reduction on Insomnia Severity Index (ISI) from baseline to 2 months	9-mo & 18-mo FU **Mean difference improvers vs. non-improvers [95% CI]:** 9mo: ISI: 3.33 (1.35, 5.31) (non-clinically important difference favouring non-improvers) SE: − 2.49% (− 8.15, 3.17%) (no statistically significant difference) PSQI: 1.05 (− 0.40, 2.50) (no statistically significant difference) PSQI-1: 0.17 (− 0.16, 0.50) (no statistically significant difference) DBAS: 1.24 (− 7.91, 10.39) (no statistically significant difference) FOSQ: − 0.2 (− 0.92, 0.52) (no statistically significant difference) FFS: 2.3 (− 1.08, 5.68) (no statistically significant difference) ESS: − 0.05 (− 1.85, 1.75) (no statistically significant difference) GCPS: 0.7 (− 0.22, 1.62) (no statistically significant difference) AIMS: − 0.83 (− 1.80, 0.14) (no statistically significant difference) PCS: 2.73 (− 0.62, 6.08) (no statistically significant difference) Tampa Scale for Kinesiophobia: 2.97 (− 1.01, 6.95) (no statistically significant difference) GDS: 1.12 (− 1.21, 3.45) (no statistically significant difference) Mean difference improvers vs. non-improvers [95% CI]: 18mo: ISI: 3.33 (1.26, 5.42) (non-clinically important difference favouring non-improvers) SE: − 2.49% (− 8.71, 3.73%) (no statistically significant difference) PSQI: 1.05 (− 0.47, 2.57) (no statistically significant difference) PSQI-1: 0.18 (− 0.17, 0.53) (no statistically significant difference) DBAS: 1.24 (− 7.91, 10.39) (no statistically significant difference) FOSQ: − 0.2 (− 0.96, 0.56) (no statistically significant difference) FFS: 2.3 (− 1.42, 6.02) (no statistically significant difference) ESS: − 0.04 (− 2.2, 1.94) (no statistically significant difference) GCPS: − 0.3 (− 1.30, 0.70) (no statistically significant difference) AIMS: − 0.83 (− 1.96, 0.30) (no statistically significant difference) PCS: 2.73 (− 0.86, 6.32) (no statistically significant difference) TSK: 2.97 (− 1.28, 7.22) (no statistically significant difference) GDS: 1.12 (− 1.42, 3.66) (no statistically significant difference) **Mean difference improvers vs. non-improvers [95% CI]:** ***Sleep and fatigue outcomes:*** ISI: − 3.03 (− 3.74, − 2.32) (non-clinically important difference favouring improvers) SE: 1.29% (− 0.18, 2.76) (no statistically significant difference) PSQI: − 1.45 (− 1.97, − 0.93) (non-clinically important difference favouring improvers) DBAS: − 2.44 (− 4.74, − 0.15) (non-clinically important difference favouring improvers) FOSQ: 0.20 (− 0.03, 0.43) (no statistically significant difference) FFS: − 1.99 (− 3.01, − 0.98) (non-clinically important difference favouring improvers) ESS: − 0.35 (− 1.00, 0.29) (no statistically significant difference) ***Pain and depression outcomes:*** GCPS: − 0.51 (− 0.80, − 0.21) (non-clinically important difference favouring improvers) AIMS: 0.63 (0.26, 1.00) (non-clinically important difference favouring non-improvers) PCS: 1.33 (− 2.94, 0.29) (no statistically significant difference) TSK: − 2.27 (− 3.95, − 0.58) (non-clinically important difference favouring improvers) GDS: − 0.52 (− 1.36, 0.32) (no statistically significant difference)
Smith et al. 2015 [32] USA RCT	Knee OA American College of Rheumatology criteria for classification of knee OA Kellgren/Lawrence Grade ≥ 1 Typical knee pain ratings ≥2 of 10 experienced > 5 days/week for > 6 months *N* = 100 79% female Mean age = 59.4y (SD 9.5)	DSM-IV-TR for insomnia Level of sleep difficulty: moderate-severe insomnia	CBT insomnia (CBT-I) Behavioral desensitization (BD) (placebo)	Sleep restriction therapy, stimulus control therapy, cognitive therapy for insomnia, sleep hygiene education Presented as means of eliminating the conditioned arousal through imagery	Individual 45 min sessions 1 x/week, for 8 weeks delivered by mental health professionals	**Sleep** ***Sleep continuity:*** all measures recorded by diary, actigraphy, or PSG (polysomnography) *Wake after sleep onset (WASO):* (lower is better) MCID: ≥0.2* *Total Sleep Time (TST):* (higher is better) MCID: ≥0.2* *Sleep-onset latency (SOL):* (lower is better) MCID: ≥0.2* *Sleep efficiency (SE):* 0–100% (higher is better) MCID: ≥0.2* *Insomnia Severity Index (ISI):* 0–28 (lower is better) MCID: ≥0.2* **Pain** ***Pain intensity:*** *Visual Analogue Scale (VAS):* 0–100 (lower is better) MCID: ≥0.2* *Western Ontario and McMaster Universities Osteoarthritis Index (WOMAC pain):* 0–10 (lower is better) MCID: ≥0.2* *Conditioned Pain Modulation (CPM):* (> 1 better) *Temporal summation:* (lower is better) MCID: ≥0.2*	Post-treatment, 3 and 6 months FU Between-group treatment effect sizes CBT-I vs. BD (cohen’s d [95%CI*]) **WASO:** ***Diary*** Posttreatment: − 0.28 (− 0.93, − 0.09) (clinically important difference favouring CBT-I) 3 mo: − 0.38 (− 1.16, − 0.21) (clinically important difference favouring CBT-I) 6 mo: − 0.15 (− 0.67, 0.27) (no statistically significant difference) ***Actigraphy*** Posttreatment: − 0.42 (− 0.83, 0.04) (no statistically significant difference) 3 mo: − 0.27 (− 0.75, 0.19) (no statistically significant difference) 6 mo: − 0.21 (− 0.57, 0.45) (no statistically significant difference) ***PSG*** Posttreatment: − 0.31 (− 1.09, − 0.21) (clinically important difference favouring CBT-I) 3 mo: 0.45 (− 0.32, 0.63) (no statistically significant difference) 6 mo: 0.00 (− 0.79, 0.16) (no statistically significant difference) **TST:** ***Diary*** Posttreatment: − 0.49 (− 1.03, − 0.18) (clinically important difference favouring BD) 3 mo: 0.01 (− 0.46, 0.46) (no statistically significant difference) 6 mo: 0.03 (− 0.56, 0.38) (no statistically significant difference) ***Actigraphy*** Posttreatment: − 0.44 (− 1.08, − 0.19) (clinically important difference favouring BD) 3 mo: 0.09 (− 0.53, 0.39) (no statistically significant difference) 6 mo: − 0.25 (− 0.99, 0.05) (no statistically significant difference) ***PSG*** Posttreatment: − 0.40 (− 0.78, 0.08) (no statistically significant difference) 3 mo: − 0.04 (− 0.47, 0.48) (no statistically significant difference) 6 mo: − 0.22 (− 0.74, 0.22) (no statistically significant difference) **SOL:** ***Diary*** Posttreatment: 0.07 (− 0.67, 0.16) (no statistically significant difference) 3 mo: − 0.15 (− 1.17, − 0.22) (non-clinically significant difference favouring CBT-I) 6 mo: 0.01 (− 0.79, 0.16) (no statistically significant difference) ***Actigraphy*** Posttreatment: 0.20 (− 0.19, 0.69) (no statistically significant difference) 3 mo: 0.24 (− 0.28, 0.65) (no statistically significant difference) 6 mo: 0.06 (− 0.52, 0.50) (no statistically significant difference) ***PSG*** Posttreatment: 0.42 (− 0.16, 0.70) (no statistically significant difference) 3 mo: − 0.09 (− 0.89, 0.07) (no statistically significant difference) 6 mo: 0.06 (− 0.53, 0.42) (no statistically significant difference) **SE:** ***Diary*** Posttreatment: 0.22 (− 0.14, 0.69) (no statistically significant difference) 3 mo: 0.39 (0.24, 1.18) (clinically important difference favouring CBT-I) 6 mo: 0.20 (− 0.12, 0.82) (no statistically significant difference) ***Actigraphy*** Posttreatment: − 0.06 (− 0.43, 0.43) (no statistically significant difference) 3 mo: 0.11 (− 0.33, 0.60) (no statistically significant difference) 6 mo: − 0.09 (− 0.65, 0.37) (no statistically significant difference) ***PSG*** Posttreatment: 0 (− 0.14, 0.72) (no statistically significant difference) 3 mo: − 0.12 (− 0.40%, 0.56) (no statistically significant difference) 6 mo: 0.01 (− 0.23, 0.73) (no statistically significant difference) **ISI:** Posttreatment: − 0.44 (− 0.86, − 0.03) (clinically important difference favouring CBT-I) 3 mo: − 0.24 (− 0.58, 0.35) (no statistically significant difference) 6 mo: − 0.62 (− 1.01, − 0.07) (clinically important difference favouring CBT-I) **VAS:** Posttreatment: 0.04 (1.45, 2.45) (non-clinically important difference favouring BD) 3 mo: − 0.17 (− 0.64, 0.25) (no statistically significant difference) 6 mo: 0.09 (− 0.43, 0.51) (no statistically significant difference) **WOMAC pain:** Posttreatment: 0.12 (− 0.44, 0.38) (no statistically significant difference) 3 mo: 0.01 (− 0.48, 0.42) (no statistically significant difference) 6 mo: 0.25 (− 0.26, 0.64) (no statistically significant difference) **CPM:** Posttreatment: − 0.19 (− 0.52, 0.33) (no statistically significant difference) 3 mo: 0.31 (− 0.04, 0.91) (no statistically significant difference) 6 mo: − 0.03 (− 0.40, 0.60) (no statistically significant difference) **Temporal summation:** Posttreatment: − 0.20 (− 0.52, 0.34) (no statistically significant difference) 3 mo: − 0.13 (− 0.45, 0.50) (no statistically significant difference) 6 mo: − 0.17 (− 0.52, 0.43) (no statistically significant difference)
Lerman et al. 2017 [43] USA Secondary analysis of RCT by Smith et al. 2015 [32]						**Pain** ***Catastrophizing:*** *Pain Catastrophizing Scale (PCS):* 0–52 (lower is better) MCID: 4.5* (defined as 30% reduction from baseline) *Daytime catastrophizing:* 0–100 (lower is better) MCID: 8.9* (defined as 30% reduction from baseline) *Nocturnal catastrophizing:* 0–100 (lower is better) MCID: 7.1* (defined as 30% reduction from baseline)	Post-treatment, 3 and 6 months FU Between-group mean differences (CBT-I vs. BD) [95% CI]* ***PCS:*** Posttreatment: 1.04 (− 3.07, 5.15) (no statistically significant difference) 3 mo: − 1.28 (− 5.43, 2.87) (no statistically significant difference) 6 mo: 0.54 (− 3.64, 4.72) (no statistically significant difference) ***Daytime catastrophizing:*** Posttreatment: 2.09 (− 4.85, 9.03) (no statistically significant difference) 3 mo: 2.75 (− 4.58, 10.08) (no statistically significant difference) 6 mo: 0.37 (− 7.11, 7.85) (no statistically significant difference) ***Nocturnal catastrophizing:*** Posttreatment: − 0.32 (− 6.82, 6.18) (no statistically significant difference) 3 mo: 0.65 (− 5.82, 7.12) (no statistically significant difference) 6 mo: − 2.73 (− 9.63, 4.17) (no statistically significant difference)
Salwen et al. 2017 [36] USA Secondary analysis of RCT by Smith et al. 2015 [32]	Knee OA American College of Rheumatology criteria for classification of knee OA. Kellgren/Lawrence Grade ≥ 1 Typical knee pain ratings ≥2 of 10 experienced > 5 days/week for > 6 months *N* = 74 77% female Mean age = 59.5y (SD 9.9)					**Sleep** ***Sleep continuity:*** *Total sleep time (TST)*: self-reported, actigraphy (higher better) MCID: 40 min* *Sleep efficiency (SE):* 0–100% (higher better) MCID: 5%* *Sleep onset latency (SOL):* (lower better) MCID: > 30 min *Wake after sleep onset (WASO):* (lower better) MCID: 30 min **Pain** *Western Ontario and McMaster Universities Osteoarthritis Index* *(WOMAC pain):* 0–10 (lower is better) MCID: 1.4* (defined as 30% reduction from baseline) Responders: reported ≥30% improvement in self-reported pain from baseline to 6 m follow-up	6 months FU Pain responders: 31/74 (42%) Nonresponders: 43/74 (58%) Mean difference mid-treatment (pain responders – nonresponders) [95% CI]*) SE: − 0.02 (− 0.06, 0.02) (no statistically significant difference) SOL: − 4.24 (− 15.13, 6.65) (no statistically significant difference) TST: − 14.67 (− 43.04, 13.70) (no statistically significant difference) WASO: 8.89 (− 6.14, 23.92) (no statistically significant difference) Patients who achieved 382 min (6 h) TST per night by mid-treatment (4 weeks) were more likely to report clinically significant pain reduction (≥30% reduction) at FU regardless of treatment group (sensitivity 54.8%, specificity 81.4%)

Notes: *CI* Confidence Interval; *FU* follow-up; *MCID* minimal clinically important difference; *mo* months; *OA* osteoarthritis; *SD* standard deviation; *vs*: versus; *y* years

*Calculated or reported by review authors

### Data synthesis and analysis

We synthesized all eligible studies qualitatively using the Synthesis without Meta-analysis (SWiM) in systematic reviews reporting guideline [28].

To quantify the effectiveness of interventions, we used the data provided in the studies to measure the association between interventions and outcomes by computing the relative risk and its 95% CI where this information was available. Similarly, we computed the difference in mean change between groups and 95% CI to quantify the effectiveness of interventions. The computation of the 95% CI for the difference in mean change is based on the assumption that the pre- and post-intervention outcomes are highly correlated (r = 0.8) [29, 30]. A meta-analysis was not conducted due to the clinical, statistical, and methodological heterogeneity of the studies. We used minimal clinically important differences (MCID) to determine clinically important between-group effects. These included: 1) a 30% change from baseline for 11 outcomes, including the Insomnia Severity Index (ISI) [31], Graded Chronic Pain Scale (GCPS) [31], Western Ontario and McMaster Universities Osteoarthritis Index (WOMAC pain) [32, 33], Arthritis Impact Measurement Scale V2 (AIMS) [33], Dysfunctional Beliefs and Attitudes About Sleep Scale (DBAS-10) [33], Flinders Fatigue Scale (FFS) [33], 10-item Functional Outcomes of Sleep Questionnaire (FOSQ-10) [33], Geriatric Depression Scale (GDS) [33], Pain Catastrophizing Scale (PCS) [32, 33], Daytime Catastrophizing [32, 33], and Nocturnal Catastrophizing [32, 33], 2) a 5% greater sleep efficiency (SE) [24], 3) a 3 point reduction for the Pittsburgh Sleep Quality Index (PSQI) [34], 4) a 2 point reduction for the 8-item Epworth Sleepiness Scale (ESS) [35], 5) a 40 min increase for Total Sleep Time (TST) [36, 37], 6) a 30 min reduction for Sleep Onset Latency (SOL) [32, 37], 7) a 30 min reduction for Wake After Sleep Onset (WASO) [32, 37], 8) a 4 point reduction for the Tampa Scale for Kinesiophobia (TSK) [38], 9) a greater than 1 point for Conditioned Pain Modulation [39], and 10) a standardized mean difference (SMD)/effect size (e.g., Cohen’s d) ≥0.2 [40]. If the MCID was unknown, we deemed a 30% between-group difference as clinically important [33]. All data were analyzed using Microsoft Excel (2007).

We categorized interventions as having a positive effect (superior to comparison group), inconclusive effect (some positive and some negative outcomes), no effect (similar outcomes to comparison), or a negative effect (inferior to comparison). The effect estimates for positive or negative effects had to be statistically and clinically significant (i.e., equal to or greater than the pre-determined MCID threshold).

## Results

### Study selection

**Fig. 1:**
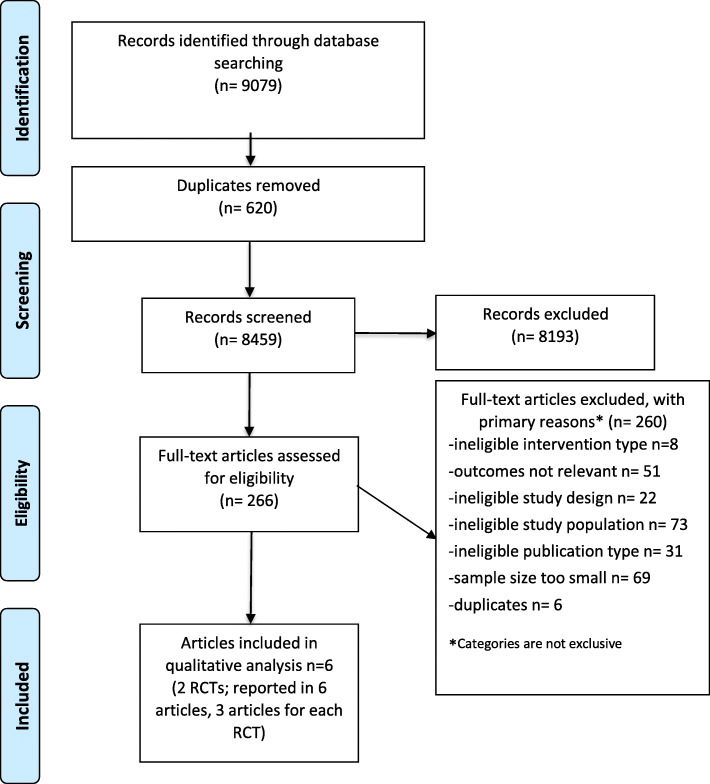
Preferred Reporting Items for Systematic Reviews and Meta-Analyses (PRISMA) Flow Diagram

We screened 8459 citations (Fig. 1). Two RCTs (reported in 6 articles; 3 articles for each RCT) were eligible and critically appraised. Both RCTs had a low risk of bias [31, 32, 36, 41–43]. We did not identify any eligible cohort or case-control studies.

### Study characteristics

Both RCTs were conducted in the U.S. and comprised of participants with OA pain and insomnia (diagnosed according to DSM-IV-TR) (Table 1) [31, 32, 36, 41–43]. Collectively, the studies analyzed 467 participants. The “Lifestyles RCT” assessed whether older persons [*n* = 367, mean age 73 years (SD 8.2), 78% female] with OA pain and insomnia receiving CBT for pain and insomnia (CBT-PI), a cognitive behavioral pain coping skills intervention (CBT-P), or an education-only control (EOC) differed in sleep and pain outcomes up to 9 months [31] and at 18 months [42]. All arms received six weekly 90-min group sessions delivered by mental health professionals. Group sizes ranged from five to 12 individuals. CBT-P included pain education, physical activation, goal setting, relaxation, activity pacing, guided imagery, and cognitive restructuring. CBP-PI added standard components of CBT-I to CBT-P (i.e., sleep hygiene education, stimulus control, sleep restriction, and daily sleep monitoring). The EOC was designed as an attention control, and included educational content related to sleep and pain management; however, classes were facilitated in a nondirective, self-help format that did not include homework assignments, guided practice in CBT principles, or daily behavioral self-monitoring. At baseline, all treatment arms had subthreshold levels of insomnia (range 11.2 (SD 5.2) -11.8 (SD 4.7) on Insomnia Severity Index [ISI], 0–28), and pain severity (range 4.1 (SD 1.5) – 4.6 (SD 1.5) on Graded Chronic Pain Scale [GCPS], 0–10). Vitiello et al. then examined the relationship between short-term (2-month) sleep improvement and long-term (9- and 18-month) sleep, pain, and fatigue outcomes [41].

The other RCT evaluated the efficacy of CBT for insomnia (CBT-I) vs. placebo (behavioral desensitization, BD) in patients with knee OA and insomnia [*n* = 100, mean age 59.4 years (SD 9.5), 79% female], to determine whether mid-treatment improvements in sleep predicted reduced pain at 3 months and 6 months [32, 36]. Both arms received eight weekly 45-min individual sessions delivered by mental health professionals. CBT-I included sleep restriction therapy, stimulus control therapy, cognitive therapy for insomnia, and sleep hygiene education. At baseline, participants had moderate severity insomnia (17 (SD 5) ISI, 0–28) and pain (5 (SD 2.1), WOMAC total score, 0–10). Lerman at al. evaluated changes in pain-catastrophizing in the sample at baseline, mid-treatment, post-treatment, 3 months and 6 months [43].

### Risk of Bias within studies

Both RCTs used appropriate randomization and blinding procedures of outcome assessors, used valid and reliable outcome measures, adjusted for differences in baseline characteristics between groups to achieve similarity at baseline, and performed intention-to-treat analyses (Table 2) [31, 32, 36, 41–43]. One RCT had follow-up rates above 80% [31, 41, 42] and the other RCT reported follow-up rates of 70% [32, 36, 43]. One weakness in both studies was that it was unclear if participants also received any interventions outside of the study.

**Table 2:** Risk of Bias Table for Randomized Controlled Trials According to the Scottish Intercollegiate Guidelines Network (SIGN) Checklist

Author, Year	Research Question	Randomization	Concealment	Blinding (1/2)	Similarity at baseline	Similarity between arms	Outcome measurement	Percent drop-out	Intention to treat	Comparable results between sites
Lerman S.F. et al. 2017 [43]	Y	Y	Y	N/Y	Y	Y	Y	CBT =30% Control =24%	Y	CS
McCurry S.M. et al. 2014 [42]	Y	Y	CS	N/Y	Y	CS	Y	CBT-Pain =13.9% CBT-Pain-Insomnia =17.2% Control =7.3%	Y	CS
Salwen J. et al., 2017 [36]	Y	Y	Y	N/Y	Y	Y	Y	Int: 30% Contr: 24%	Y	N/A
Smith M.T., et al. 2015 [32]	Y	Y	Y	N/Y	Y	Y	Y	CBT =30% Control =24%	Y	N/A
Vitiello et al. 2014 [41]	Y	Y	CS	N/Y	Y	CS	Y	CBT-Pain: 3.3% CBT-Pain & Insomnia: 6.6% Control: 0.8%	Y	CS
Vitiello et al. 2013 [31]	Y	Y	CS	N/Y	Y	CS	Y	CBT-Pain: 9.0% CBT-Pain & Insomnia: 11.4% Control: 2.4%	Y	CS

*Y* Yes; *N* No; *CS* Can’t Say; *N/A* Not applicable. *CBT* cognitive behavioural therapy

1. Blinding of subjects and/or treatment providers; 2. Blinding of outcome assessors/data analysists

### Summary of evidence

The Lifestyles trial assessed older patients with comorbid OA pain and insomnia at 9 and 18 months. At 9 months, CBT-PI (vs. education only) provided clinically important improvements in self-reported sleep (measured by Insomnia Severity Index [ISI]; OR 2.20, 95% CI 1.25, 3.90)) [31]. CBT-PI (vs. CBT-P) also provided clinically important improvements in self-reported sleep (measured by ISI) in all participants (OR 2.72, 95% CI 1.59, 4.64) and in a subgroup of patients with severe pain at baseline (OR 3.21, 95% CI 1.22, 8.43). CBT-P (vs. education only) provided clinically important improvements in sleep efficiency (measured by wrist actigraphy) in a subgroup of participants with severe pain at baseline (mean difference 5.45, 95% CI 1.56, 9.33) [31]. At 18 months, CBT-PI, CBT-P, and education had similar effectiveness on sleep outcomes (i.e., insomnia severity, sleep efficiency) [42]. These interventions also had similar effectiveness on pain outcomes (measured by Graded Chronic Pain Scale [GCPS], Arthritis Impact Measurement Scale [AIMS]) at 9 and 18 months [31, 42]. Across all intervention arms, short-term (2-month) clinically significant improvements in sleep were not associated with long-term (9- and 18-month) clinically significant improvements in any of the sleep outcomes (ISI, SE, Pittsburgh Sleep Quality Index [PSQI], Dysfunction Beliefs and Attitudes About Sleep [DBAS] scale, Flinders Fatigue Scale [FFS]), Epworth Sleepiness Scale [ESS], Functional Outcomes of Sleep Questionnaire [FOSQ]) or pain or health outcomes (AIMS, GCPS, Pain Catastrophizing Scale [PCS], Tampa Scale for Kinesiophobia, Geriatric Depression Scale [GDS]).

Smith et al. assessed patients with comorbid knee OA and insomnia at 2, 3 and 6 months.(48) They found that compared to placebo (behavioral desensitization), patients in the CBT-I group improved in: 1) wake after sleep onset (WASO) measured by diary at post-treatment (2 months) (Cohen’s d − 0.28, 95% CI -0.93, − 0.09) and 3 months (Cohen’s d − 0.38, 95% CI -1.16, − 0.21), and by polysonography (PSG) at post-treatment (Cohen’s d − 0.31, 95% CI -1.09, − 0.21); 2) total sleep time (TST) at post-treatment measured by diary (Cohen’s d − 0.49, 95% CI -1.03, − 0.18) and actigraphy (Cohen’s d − 0.44, 95% CI -1.08, − 0.19); 3) SE measured by diary at 3 months (Cohen’s d 0.39, 95% CI 0.24, 1.18); and 4) ISI measured at post-treatment (Cohen’s d − 0.44, 95% CI -0.86, − 0.03) and 6 months (Cohen’s d − 0.62, 95% CI -1.01, − 0.07). The intervention was no better than placebo for improving sleep onset latency (SOL) or pain outcomes (VAS, Western Ontario and McMaster Universities Arthritis Index [WOMAC] pain subscale, Conditioned Pain Modulation, Temporal Summation) at any time point. In a subsequent analysis, achieving approximately 6 h of total sleep time (TST) (measured by self-report) in any treatment arm by 4 weeks predicted clinically significant pain reduction at 6 months (WOMAC pain subscale) (sensitivity 54.8%, specificity 81.4%) [36]. In another secondary analysis, CBT-I was no better than placebo in reducing pain-catastrophizing (Pain Catastrophizing Subscale [PCS]) [43].

## Discussion

### Summary of evidence

We identified two low risk of bias RCTs (reported in 6 articles; 3 articles for each RCT) assessing the effectiveness of CBT on sleep and pain outcomes in adults with OA and comorbid insomnia. Both RCTs had similar findings. CBT for insomnia (CBT-I) either alone [32] or combined with CBT for pain (CBT-PI) [31, 42] improved some sleep outcomes (sleep efficiency, characteristics related to sleep-onset) at 9 months [32] but not at 18 months [42]. Intervention and comparison groups had similar effects on pain outcomes at all follow-up points. There was one inconsistent finding between the RCTs. Salwen et al. [36] found that achieving clinically significant improvement in sleep in the short term was associated with clinically significant improvement in pain at 6 months; however, Vitiello et al. (2014) [41] found no improvements. This inconsistency may be explained, in part, by differences in study population, interventions, comparisons and follow-up periods. The participants in Smith et al. were younger, had greater severity of insomnia and pain at baseline, used a placebo comparison group as opposed to active comparison groups, and followed up participants at 6 months as compared to 9 and 18 months [41].

Trial authors discussed why CBT-I or CBT-PI may not have been shown to be superior to comparison groups at improving pain in people with comorbid insomnia. One potential reason is that a number of patients that entered the Lifestyles trial had subclinical levels of insomnia and pain, therefore reducing the potential for detecting improvement in these outcomes (reaching MCID thresholds) [31, 41, 42]. Baseline severity may need to be above some minimal threshold for reciprocal and durable effects of treating sleep and pain to be observed; for example, moderate insomnia (ISI score: 15–21) [44] and moderate pain-related disability (CPGS Grade III) [45]. To better understand why interventions did or did not work or were delivered as intended, all interventions, comparison interventions (including sham and control groups), and intervention components should be explicitly described. Using the template for intervention description and replication (TIDieR) checklist may facilitate this [46]. This is especially important for complex interventions consisting of various therapeutic components, such as CBT, which may be delivered in various ways and with various intensities. Indeed, authors in both trials in our review explained that the comparison interventions may have also had effective components on pain (i.e., CBT-P, education, and behavioral desensitization), thereby reducing the potential for detecting intervention effects. A comprehensive description of the interventions may have facilitated that assessment.

### Other systematic reviews

Our results are consistent with other systematic reviews reporting that non-pharmacological sleep interventions are promising for people with pain conditions; however, authors also suggested that further research is needed. For example, Afolalu et al. (2018) found that changes in sleep are prospectively associated with pain-related outcomes [47]. Ho et al. (2019) found that CBT and pharmacological interventions appeared to improve sleep and pain for people with LBP and sleep for people with OA [19]. Our review captured the same RCTs assessing individuals with OA; however, we did not identify information on low back pain because we excluded studies with small sample size and those assessing pharmacological interventions. Ho et al. explained that while improvements in pain were below the MCID of 15/100 (VAS) for people with chronic low back pain, they considered the improvement important as most interventions occurred for 6–8 weeks, and OA pain worsens over time [19]. Conditions such as low back pain are self-limiting and often cyclical in nature compared to the exacerbating trajectory of OA [48, 49]. The nature of the MSK condition should be considered when determining effect over a variety of follow-up times as this may impact the ability to recognize effect. With respect to Ho et al. (2019), observing clinically important changes in pain may have been inhibited by the design of the included studies (small sample sizes and did not restrict individuals with comorbid sleep conditions at baseline). Finally, Tang et al. (2015) found that non-pharmacological sleep interventions (e.g., education, CBT) represent a promising avenue for optimizing treatment outcomes in patients with chronic cancer and non-cancer pain conditions [18].

### Strengths and limitations

Strengths of our systematic review included a comprehensive literature search strategy that was peer-reviewed by a second health sciences librarian using the PRESS Checklist [26]. We conducted and reported our systematic review according to the PRISMA statement [21] and used explicit criteria for independent reviewers to conduct screening, critical appraisal, and data extraction. We used MCID thresholds to determine clinically important between-group effects. MCIDs contribute to the interpretation of the outcomes indicating whether the effects of an intervention are clinically meaningful. Determining accurate MCIDs for specific populations is often challenging as the literature in this area is scarce. While our aim was to determine the most suitable MCID for each outcome measure, population and context, it is possible that having selected alternate MCIDs may have led to varying results. However, we reported all the effect sizes and MCIDS; allowing readers to interpret the results and determine clinical importance. This review has limitations. First, we only included studies published in English to increase feasibility, which may have excluded relevant studies published in other languages, however, this is an unlikely source of bias [50–54]. Second, we only included published peer-reviewed studies; therefore, we were unable to assess for potential publication bias. Third, studies had to include a sleep-related outcome as per the inclusion criteria to be considered relevant. Therefore, we may have excluded studies that tested the effectiveness of interventions directed at sleep problems based on pain or other outcomes, but did not include a sleep-related outcome. However, as our research question assesses the effectiveness of these interventions on sleep characteristics, these studies would be outside the scope of our review.

### Clinical implications

Despite its high prevalence and burden, sleep problems (apart from severe sleep disorders frequently requiring pharmacological or other medical treatment e.g., sleep apnea, narcolepsy, and sleep terrors) are often unrecognized and left untreated because of barriers to assessment and management [55, 56]. Given the strong bi-directional relationship between sleep and pain [2, 57], addressing sleep issues early on in the care plan and taking a more pro-active approach in sleep treatment may be beneficial for optimizing treatment outcomes in patients living with chronic MSK painful conditions and comorbid sleep problems. While our review is limited in providing solutions long-term, clinical guidelines recommend screening and education as it is low-cost and non-invasive as it may provide benefit to patients while further research is conducted [58].

### Future research

Only two RCTs were identified as relevant in our review. More high-quality research, particularly RCTs, are needed focusing on other non-pharmacological interventions in individuals with comorbid sleep problems and other MSK conditions, with varying degrees of pain, in addition to OA. To determine the enduring effects of non-pharmacological treatments in improving both sleep and pain, future research may need to target individuals with more severe and persistent insomnia and pain symptoms. Furthermore, the duration of the intervention should be consistent. In Vitiello et al., both CBT-I and CBT-PI intervention were 90 min in duration suggesting that some of the insomnia content in the combined CBT-PI may have been briefer than other CBT-I treatments. While CBT-I is well-established and it offers treatment components that are based on known physiological mechanisms underlying sleep, the mechanisms for chronic pain are not as well understood making this important to further explore. In addition to sleep-related outcomes, investigators should select other outcomes important to individuals with painful MSK conditions, such as those related to improved function and participation in meaningful life activities. It is possible that improved sleep may help improve individuals’ self-efficacy, function and participation despite having similar levels of pain intensity. Future trials should assess the effectiveness of combining first line treatments for sleep problems (e.g., CBT-I) with first line treatments for MSK pain (e.g., reassurance, education, exercise, manual therapy). It is possible that this combination may augment their beneficial effects on sleep and pain.

## Conclusion

Our review demonstrated that CBT-I or CBT-PI provide some benefits to improving sleep outcomes, but not pain or other health outcomes, in adults with comorbid insomnia and osteoarthritis. Further high-quality research is needed, particularly on other non-pharmacological interventions for comorbid sleep problems and a range of MSK conditions. In addition, further high-quality research is required to determine if sleep-focused treatments, such as CBT-I, targeted at people with comorbid sleep problems and other MSK conditions, is effective at improving their sleep and pain.

## Supplementary Information

**Additional file 1.**

## Data Availability

The datasets used and/or analyzed during the current study are available from the corresponding author on reasonable request.
