# Supplementary material for: Effectiveness of non-pharmacological interventions on sleep characteristics among adults with musculoskeletal pain and a comorbid sleep problem: a systematic review
Source: Chiropr Man Therap. 2021 Jul 8;29:23. doi: 10.1186/s12998-021-00381-6 (PMC8268365; doi:10.1186/s12998-021-00381-6)
Supplement: Supplementary file 1 — Additional file 1. [file 12998_2021_381_MOESM1_ESM.docx]

MEDLINE Search Strategy

MEDLINE: Epub Ahead of Print, In-Process & Other Non-Indexed Citations, Ovid MEDLINE® Daily and Ovid MEDLINE® 1946-Present; inception to April 2, 2020

1. Sleep/
2. Sleep Deprivation/
3. Sleep Disorders/
4. "Sleep Initiation and Maintenance Disorders"/
5. Sleep Stages/
6. exp Fatigue/
7. Biological Clocks/
8. Wakefulness/
9. (sleep* adj4 (aids or alteration* or chronotype* or complaint* or debt or deprivation or deprived or disorder* or disturbance* or duration or education or environment or habit* or health or hygiene or outcome* or parameter* or pattern* or poor or problem* or quality or schedul* or status or symptom* or variable*)).ab,ti.
10. alertness.ab,ti.
11. "awakening*".ab,ti.
12. "sleepiness or somnolence".ab,ti.
13. Insomnia.ab,ti.
14. or/1-13 [**sleep]
15. Whiplash Injuries/
16. Neck Injuries/
17. Neck Pain/
18. Neck Muscles/in [Injuries]
19. exp Cervical Vertebrae/in [Injuries]
20. Radiculopathy/
21. exp Brachial Plexus Neuropathies/
22. exp Torticollis/
23. whiplash.ab,ti.
24. "neck injur*".ab,ti.
25. "neck pain*".ab,ti.
26. "neck ache*".ab,ti.
27. "neckache*".ab,ti.
28. "brachial plexus neuropath*".ab,ti.
29. torticollis.ab,ti.
30. or/14-29 [**neck pain]
31. exp Back Injuries/
32. exp Back Pain/
33. Coccyx/in [Injuries]
34. Intervertebral Disc Degeneration/
35. Intervertebral Disc Displacement/
36. Lumbar Vertebrae/in [Injuries]
37. Lumbosacral Region/in [Injuries]
38. Osteoarthritis, Spine/
39. Piriformis Muscle Syndrome/
40. Sciatica/
41. Spinal Diseases/
42. Spinal Stenosis/
43. (back adj3 (ache* or injur* or pain*)).ab,ti.
44. (backache* adj3 (injur* or pain*)).ab,ti.
45. (back pain or back-pain).ab,ti.
46. (lumbar disc* adj3 (extruded or degenerat* or herniat* or prolapse* or sequestered or slipped)).ab,ti.
47. (lumbar disk* adj3 (extruded or degenerat* or herniat* or prolapse* or sequestered or slipped)).ab,ti.
48. "low* back pain".ab,ti.
49. (lumbar adj3 (pain or facet or nerve root* or osteoarthritis or radicul* or spinal stenosis or spondylo* or zygapophys*)).ab,ti.
50. "Piriformis syndrome*".ab,ti.
51. (sacral adj2 pain*).ab,ti.
52. ((spine or spinal) adj4 (condition* or disable* or disabilit* or disorder* or pain or stenos?s)).ab,ti.
53. spondylosis.ab,ti.
54. or/30-53 [**back pain]
55. Thoracic Injuries/
56. Rib Fractures/
57. Intercostal Muscles/
58. Chest Pain/
59. Angina Pectoris/
60. Microvascular Angina/
61. Tietze's Syndrome/
62. (thoracic adj4 (spine or spinal)).ab,ti.
63. (T-spine or T-spinal).ab,ti.
64. thoracic wall.ab,ti.
65. "thoracic injur*".ab,ti.
66. "chest adj4 pain*".ab,ti.
67. cervicothoracic angina.ab,ti.
68. angina pectoris.ab,ti.
69. intercostal myalgia.ab,ti.
70. segmental thoracic dysfunction.ab,ti.
71. pectoral myalgia.ab,ti.
72. costovertebral dysfunction.ab,ti.
73. cervical angina.ab,ti.
74. fibrositis.ab,ti.
75. (costal chondritis or costochondritis or costal chondritides).ab,ti.
76. chest wall tenderness.ab,ti.
77. "slipping rib*".ab,ti.
78. "Tietze* syndrome".ab,ti.
79. sternoclavicular.ab,ti.
80. thoracolumbar.ab,ti.
81. or/54-80 [**thoracic pain]
82. Shoulder Pain/
83. exp Cumulative Trauma Disorders/
84. exp Median Neuropathy/
85. Shoulder Impingement Syndrome/
86. Shoulder Joint/in [Injuries]
87. Shoulder/in [Injuries]
88. exp Arm Injuries/
89. exp Hand Injuries/
90. Wrist Injuries/
91. Finger Injuries/
92. exp Tendinopathy/
93. Radial Neuropathy/
94. exp Ulnar Neuropathies/
95. Bursitis/
96. Thoracic Outlet Syndrome/
97. carpal tunnel syndrome.ab,ti.
98. (medial and (epicondylitis or epicondylosis or epicondylopathy)).ab,ti.
99. (lateral and (epicondylitis or epicondylosis or epicondylopathy)).ab,ti.
100. (shoulder* and (pain* or sprain* or strain* or injur* or impair* or impingement)).ab,ti.
101. (shoulder* and (tendinopathy or tendinitis or tendonitis or capsulitis)).ab,ti.
102. ((glenohumeral or scapul* or acromioclavicular) and (pain* or sprain* or strain* or injur*)).ab,ti.
103. (rotator cuff and (sprain* or strain* or tear* or bursitis tendinitis or impingement)).ab,ti.
104. ((supraspinatus or infraspinatus or subscapularis or teres minor or teres major or trapezius or deltoid or bicep* or bicipital or coracobrachialis) and (impingement or strain* or tear* or pain*)).ab,ti.
105. biceps tend?nitis.ab,ti.
106. painful arc.ab,ti.
107. frozen shoulder.ab,ti.
108. (shoulder and capsul* and (sprain* or tear*)).ab,ti.
109. (forearm* and (pain* or sprain* or strain* or injur* or impair*)).ab,ti.
110. (arm* and (pain* or sprain* or strain* or injur* or impair*)).ab,ti.
111. (wrist* and (pain* or sprain* or strain* or injur* or impair*)).ab,ti.
112. (hand* and (pain* or sprain* or strain* or injur* or impair*)).ab,ti.
113. (elbow* and (pain* or sprain* or strain* or injur* or impair*)).ab,ti.
114. "thoracic outlet syndrome*".ab,ti.
115. tennis elbow.ab,ti.
116. (rotator cuff and (injur* or disorder*)).ab,ti.
117. (median adj neuropath*).ab,ti.
118. (radial adj neuropath*).ab,ti.
119. bursitis.ab,ti.
120. "upper extremit* injur*".ab,ti.
121. ((radial or ulnar) adj neuropath*).ab,ti.
122. "cumulative trauma disorder*".ab,ti.
123. (repetit* and (strain* or sprain* or injur* or disorder*)).ab,ti.
124. or/81-123 [**upper extremity]
125. exp Hip Injuries/
126. exp Leg Injuries/
127. exp Knee Injuries/
128. exp Foot Injuries/
129. exp Toes/in [Injuries]
130. Ankle Injuries/
131. Lateral Ligament, Ankle/in [Injuries]
132. Fasciitis, Plantar/
133. (lower and (extremit* or limb* or injur*)).ab,ti.
134. (ankle* and (sprain* or strain* or injur*)).ab,ti.
135. ((talofibular or calcaneofibular or calcaneotibial or tibio*) and (sprain* or strain* or injur*)).ab,ti.
136. (buttock* and (injur* or pain*)).ab,ti.
137. (foot and (injur* or pain*)).ab,ti.
138. (hip* and (injur* or pain*)).ab,ti.
139. (knee* and (injur* or pain*)).ab,ti.
140. (leg* and (injur* or pain*)).ab,ti.
141. (thigh* and (injur* or pain*)).ab,ti.
142. (toe* and (injur* or pain* or turf)).ab,ti.
143. "patellofemoral pain syndrome*".ab,ti.
144. tendinosis.ab,ti.
145. tendinopathy.ab,ti.
146. plantar fasciitis.ab,ti.
147. or/124-146 [**lower extremity]
148. Musculoskeletal Pain/
149. ((musculoskeletal or musculo-skeletal or MSK) adj4 (care or condition* or disabilit* or disorder* or injur* or pain* or problem* or trouble*)).ab,ti.
150. 147 or 149 [**MSK pain general]
151. exp Headache Disorders/
152. Headache/
153. Post-Traumatic Headache/
154. Tension-Type Headache/
155. (headache* adj4 (tension type or tension-type or tension or muscle contraction or psychomyogenic or stress or essential or ideopathic or psychogenic or daily-persistent)).ab,ti.
156. (headache* adj4 (whiplash or WAD)).ab,ti.
157. (headache* adj4 (post traumatic or post-traumatic or PTH)).ab,ti.
158. (headache* adj4 (cervicogenic or cervical or neck or pericranial)).ab,ti.
159. or/150-158 [**headache]
160. exp Temporomandibular Joint Disorders/
161. exp Temporomandibular Joint/
162. exp Jaw/in [Injuries]
163. Mastication/
164. "costen*".ab,ti.
165. cranio mandibular.ab,ti.
166. craniomandibular.ab,ti.
167. (myofasc* and (digastricus or masseter or pterygoid or stylohyoideus or temporalis)).ab,ti.
168. ((disc or disk) and derangement*).ab,ti.
169. discus articularis.ab,ti.
170. hyoid.ab,ti.
171. (jaw* and (injur* or problem* or pain* or condition* or disorder* or syndrome* or dysfunction* or disfunction*)).ab,ti.
172. "mandible*".ab,ti.
173. mandibular.ab,ti.
174. "occlus*".ab,ti.
175. open lock.ab,ti.
176. orofacial.ab,ti.
177. "synovitis and jaw*".ab,ti.
178. temporo mandibular.ab,ti.
179. temporomandibular.ab,ti.
180. TMD.ab,ti.
181. TMJ.ab,ti.
182. or/159-181 [**TMJ]
183. 30 or 54 or 81 or 124 or 147 or 150 or 159 or 182 [**all MSK]
184. Case-Control Studies/
185. Cohort Studies/
186. Controlled Clinical Trials as Topic/
187. Epidemiologic Studies/
188. Epidemiology/
189. Follow-Up Studies/
190. Longitudinal Studies/
191. Prospective Studies/
192. Retrospective Studies/
193. Randomized Controlled Trials as Topic/
194. ((case control or case-control) adj3 (stud* or design*)).ab,ti.
195. (cohort adj3 (stud* or design* or analysis)).ab,ti.
196. controlled clinical trial.pt.
197. "epidemiolog*".ab,ti.
198. ((followup or follow-up) adj3 (stud* or design*)).ab,ti.
199. (longitudinal* adj3 (stud* or design*)).ab,ti.
200. (prospective adj3 (stud* or design*)).ab,ti.
201. (random* and (control* or clinical or allocat*)).ab,ti.
202. randomized controlled trial.pt.
203. (retrospective adj3 (stud* or design*)).ab,ti.
204. or/183-203
205. 14 and 183 and 204
206. limit 205 to english language
